# Changed Patterns of Genomic Variation Following Recent Domestication: Selection Sweeps in Farmed Atlantic Salmon

**DOI:** 10.3389/fgene.2020.00264

**Published:** 2020-04-03

**Authors:** Marina Naval-Sanchez, Sean McWilliam, Bradley Evans, José M. Yáñez, Ross D. Houston, James W. Kijas

**Affiliations:** ^1^CSIRO Agriculture and Food, Brisbane, QLD, Australia; ^2^Salmon Enterprises of Tasmania Pty. Limited, Wayatinah, TAS, Australia; ^3^Faculty of Veterinary and Animal Sciences, University of Chile, Santiago, Chile; ^4^The Roslin Institute and Royal (Dick) School of Veterinary Studies, University of Edinburgh, Midlothian, United Kingdom

**Keywords:** Atlantic salmon, selection sweep, domestication, evolution, whole genome sequence

## Abstract

The introduction of wild Atlantic salmon into captivity, and their subsequent artificial selection for production traits, has caused phenotypic differences between domesticated fish and their wild counterparts. Identification of regions of the genome underling these changes offers the promise of characterizing the early biological consequences of domestication. In the current study, we sequenced a population of farmed European Atlantic salmon and compared the observed patterns of SNP variation to those found in conspecific wild populations. This identified 139 genomic regions that contained significantly elevated SNP homozygosity in farmed fish when compared to their wild counterparts. The most extreme was adjacent to *versican*, a gene involved in control of neural crest cell migration. To control for false positive signals, a second and independent dataset of farmed and wild European Atlantic salmon was assessed using the same methodology. A total of 81 outlier regions detected in the first dataset showed significantly reduced homozygosity within the second one, strongly suggesting the genomic regions identified are enriched for true selection sweeps. Examination of the associated genes identified a number previously characterized as targets of selection in other domestic species and that have roles in development, behavior and olfactory system. These include *arcvf*, *sema6, errb4, id2-like*, and *6n1-like* genes. Finally, we searched for evidence of parallel sweeps using a farmed population of North American origin. This failed to detect a convincing overlap to the putative sweeps present in European populations, suggesting the factors that drive patterns of variation under domestication and early artificial selection were largely independent. This is the first analysis on domestication of aquaculture species exploiting whole-genome sequence data and resulted in the identification of sweeps common to multiple independent populations of farmed European Atlantic salmon.

## Introduction

Comparison between domestic animals and their wild counterparts reveals a spectrum of fundamental phenotypic and behavioral differences that includes body conformation, reproductive seasonality and life history traits. The genomic consequences of domestication, whereby previously wild populations are progressively brought under captive management and controlled breeding, has been the subject of evolutionary investigation in dog (*Canis familiaris*) (Axelsson et al., 2013; Wang et al., 2013), pig (*Sus scrofa*) (Larson et al., 2005; Frantz et al., 2015), sheep (*Ovis aries*) (Naval-Sanchez et al., 2018), sheep and goat (Alberto et al., 2018), chicken (*Gallus gallus*) (Xiang et al., 2014), rabbit (*Oryctolagus cuniculus*) (Carneiro et al., 2014), and several other species (Koenig et al., 2013; Lemmon et al., 2014). Most mammalian domesticates have a population history spanning 10,000 years or more, creating significant challenges to distinguishing evolutionary events that arose early during domestication and selection from those caused during subsequent strong positive selection and the formation of breeds. This prompted us to explore the genomic consequence of domestication in farmed Atlantic salmon (*Salmo salar*) for three key reasons. Firstly, the onset of commercial Atlantic salmon farming for human consumption commenced as recently as the 1960s in Norway. This offers the opportunity to access the early evolutionary consequences of domestication. Since the 1960’s, the industry has expanded rapidly and marked phenotypic differences now exist between farmed and wild Atlantic Salmon. These differences take many forms as extensively reviewed by Glover et al. (2017), with the two largest and most consistently observed being growth rate and survival in the wild (Fleming et al., 2000; McGinnity et al., 2003; Glover et al., 2009, 2017; Skaala et al., 2012, 2019; Solberg et al., 2013). Other differences include disease resistance, age at sexual maturation and product quality traits (Gjedrem et al., 2012; Gutierrez et al., 2014, 2016). Secondly, both European and North America wild Atlantic salmon have been independently domesticated to initiate a range of breeding programs around the world. A comparison of selection sweeps in farmed animals of European and North American origin offers the opportunity to identify genes and gene networks potentially involved in the phenotypic changes common to both farmed populations. Finally, the identification of genetic changes underlying aquaculture-associated phenotypes offers the opportunity to identify DNA markers with potential for use in selective breeding to expedite improvement of key production traits, and to understand how domestication meditated changes in escaped farmed animals may impact the population dynamics of wild populations (Glover et al., 2013; Wringe et al., 2018). Domestication-associated genetic changes are not well understood in aquaculture species. In Atlantic salmon, several studies have compared aquaculture strains against wild populations (Bourret et al., 2011; Vasemägi et al., 2012; Gutierrez et al., 2014; Mäkinen et al., 2015; Liu et al., 2017; López et al., 2019a). These have used SNP arrays that generate genome-wide allele frequency data for tens or hundreds of thousands of loci. Comparison of the outlier loci identified between studies has shown little or no overlap. This may change as additional studies emerge, and as the size of the populations under investigation and the scale and precision of genomic datasets improve.

In the current study, we utilized whole-genome sequence data obtained from populations of both farmed and wild European salmon to call SNPs and search for evidence of selection sweeps associated with domestication. Genomic regions that displayed low heterozygosity were identified in each of the groups, and a number were subsequently identified using independent populations of salmon. This increased the likelihood they reflect real selection events as opposed to neutral evolutionary forces such as random drift. Genes within regions under selection were involved in neurogenesis or previously associated with domestication in other species.

## Materials and Methods

### Samples and Sequencing

Three datasets containing genome sequence from wild and domestic Atlantic salmon were used of either European or North American origin (Table 1). Dataset 1 contained whole genome sequence derived from four pools of DNA sampled from a commercial breeding program (Landcatch, United Kingdom). Each of the four pools were created by mixing individually extracted genomic DNA from 20 to 22 individuals at equimolar concentrations, with the individuals being sampled from two different year classes of the breeding program. Each pool was sequenced (pool-seq) to a depth of 20 – 24 fold coverage using paired-end libraries with an Illumina HiSeq 2500 (Edinburgh Genomics, United Kingdom). The origin of this farmed population is composed of a mixture of wild fish from a range of Scottish rivers and introductions from Norway, including from the Norwegian river Namsen and the Mowi program. The population underwent approximately 10 generations of domestication and selection prior to sampling and sequencing. Dataset 1 also contained pool-seq data from European derived wild Atlantic salmon from previously published work (Ayllon et al., 2015, Table 1). The fastq files from six pools, each containing 20 individuals from a different river of Western Norway, were downloaded from the NCBI SRA (Bioproject number PRJNA293012). Dataset 2 sought to mirror Dataset 1 in that it contained both wild and domesticated European derived Atlantic salmon. Dataset 2 differed, however, in that it comprised individually sequenced genomes rather than pooled data (Table 1). The farmed population in Dataset 2 included fastq files of individual whole-genome sequences from 13 Chilean animals originating either from Scotland (Lochy and Landcatch strains) or Norway (Fanad and Mowi strains) (SRA deposition SRP059652) (Yáñez et al., 2016). Briefly, each individual was sequenced to a depth of 6 fold coverage using 100 bp paired-end libraries with an Illumina HiSeq 2000 machine (Macrogen, South Korea). The wild population comprised published fastq files of individual whole-genome sequences from 12 wild salmon from the Norwegian Atlantic clade rivers - Jølstra (3), Naustra (3), Namsen (3) and Argadsvassdraget (3) downloaded from SRA (Bioproject PRJEB10744) (Barson et al., 2015). Dataset 3 contained domesticated animals of North America origin, farmed in Tasmania, Australia (Kijas et al., 2018). A total of 19 animals from the SALTAS breeding program from different year classes and sex lineages were downloaded from NCBI Bio Project ID PRJNA403334, with individual animal raw sequence accession numbers SRR6019467 – SRR6019464.

**TABLE 1:** Origin of the whole genome sequences used.

**Dataset**	**Origin**	**Type**	**Data**	**Population**	**n**	**Sex**	**References**
1	EU	Farmed	Pool	Scotland	4*22	M/F	This Study
	EU	Wild	Pool	Eidselva	4*20	M	Ayllon et al., 2015
	EU	Wild	Pool	Aardselva	4*20	M	Ayllon et al., 2015
	EU	Wild	Pool	Flekke	4*20	M	Ayllon et al., 2015
	EU	Wild	Pool	Gloppenelva	4*20	M	Ayllon et al., 2015
	EU	Wild	Pool	Sulsdagen	4*20	M	Ayllon et al., 2015
	EU	Wild	Pool	Vorma	4*20	M	Ayllon et al., 2015
2	EU	Farmed	Indiv	Chile	13	M/F	Yáñez et al., 2016
	EU	Wild	Indiv	Jølstra	3	2M/1F	Barson et al., 2015
	EU	Wild	Indiv	Naustra	3	2M/1F	Barson et al., 2015
	EU	Wild	Indiv	Namsen	3	2M/1F	Barson et al., 2015
	EU	Wild	Indiv	Argadsvassdraget	3	2M/1F	Barson et al., 2015
3	NA	Farmed	Indiv	Tasmanian	20	M/F	Kijas et al., 2018

*Dataset 1 consisted of pooled samples. Dataset 2 and 3 consisted of samples sequenced individually. EU: European origin. NA, North American origin.*

### Mapping, Variant Calling, Annotation and PCA

Variant calling was performed independently for each Dataset (1 – 3) using the same pipeline and parameters. Sequence reads were first mapped to the salmon reference genome ICSASG_v2 (Lien et al., 2016) using Burrows–Wheeler algorithm (bwa) (Li and Durbin, 2009), with default parameters. Duplicate reads were removed using Picard tools^1^ and local realignment around INDELS was performed in accordance to GATK best practices (DePristo et al., 2011). Variants were called using GATK haplotypeCaller (v3.7) to produce joint genotyping calls within each dataset. Variants were filtered using bcftools filter (version 1.3.1) to remove variants (i) with mapping quality < 50 (ii) read depth < 5 and (iii) variants other than biallelic SNPs. Only variants with genotype calls in more than 90% of the samples per set were retained. The variant effect predictor tool from ensembl (version 78) was used to identify 24 separate SNP classifications in relation to the gene model annotated reference assembly ICSASG_v2 (Lien et al., 2016). Classifications included coding, missense and non-synonymous substitutions, intronic and intergenic variants. In preparation for principal component analysis (PCA), SNP subsets were identified by removal of loci in linkage disequilibrium using the –indep-pairwise 500 100 0.9 command implemented in PLINK v1.9^2^ (Chang et al., 2015). This assessed genomic bins containing 500 SNP with a 100 SNP set size and removed loci with squared correlation exceeding 0.9. PCA was also performed using PLINK v1.9 before the eigenvector values were visualized by plotting in R v3.1.2.

### Selection Sweep Detection

To identify signatures of selection in sequence data we calculated a window based heterozygosity score *H*_p_ as in Rubin et al. (2010, 2012). In brief, we determined the number of reads corresponding to the most and least abundant allele (*n*_MAJ_ and *n*_MIN_) per SNP separately for farmed and wild fish populations. The *H*_p_ metric was estimated in 150 kb genome windows with 75 kb overlap, before windows containing less than 20 SNP were discarded. For each window we calculated *H*_p_ = 2Σ*n*_MAJ_Σ*n*_MIN_/(Σ*n*_MAJ_ + Σ*n*_MIN_)^2^, where Σ*n*_MAJ_ and Σ*n*_MIN_ are the sums of *n*_MAJ_ and, respectively, *n*_MIN_ for all SNPs in the window. Individual *H*_p_ values were then Z-transformed as follows: *ZH*_p_ = (*H*_p_ - μ*H*_p_)/σ*H*_p_. To derive directionality of selection intensity, we calculated Δ*ZH*p = *ZH*p_*Wild*_ – *ZH*p_*Farmed*_. Positive Δ*ZH*p values reflect genome regions containing loss of heterozygosity in farmed fish when compared to wild fish, consistent with positive selection post domestication. Multiple testing was performed by Bonferroni correction to account for evaluation of 150 Kb genome bins with a step size of 75 Kb. A second method to detect selection sweeps exploited allele frequency differences between populations measured as *F*_*ST*_ (reviewed by Luikart et al., 2003). *F*_*ST*_ was calculated using the same 150 kb windows as *H*_p_ and compared farmed and wild populations in accordance to the Cockerham and Weir (1984) implemented in vcftools (Danecek et al., 2011).

## Results

### Genome Sequencing and SNP Calling

**FIGURE 1:**
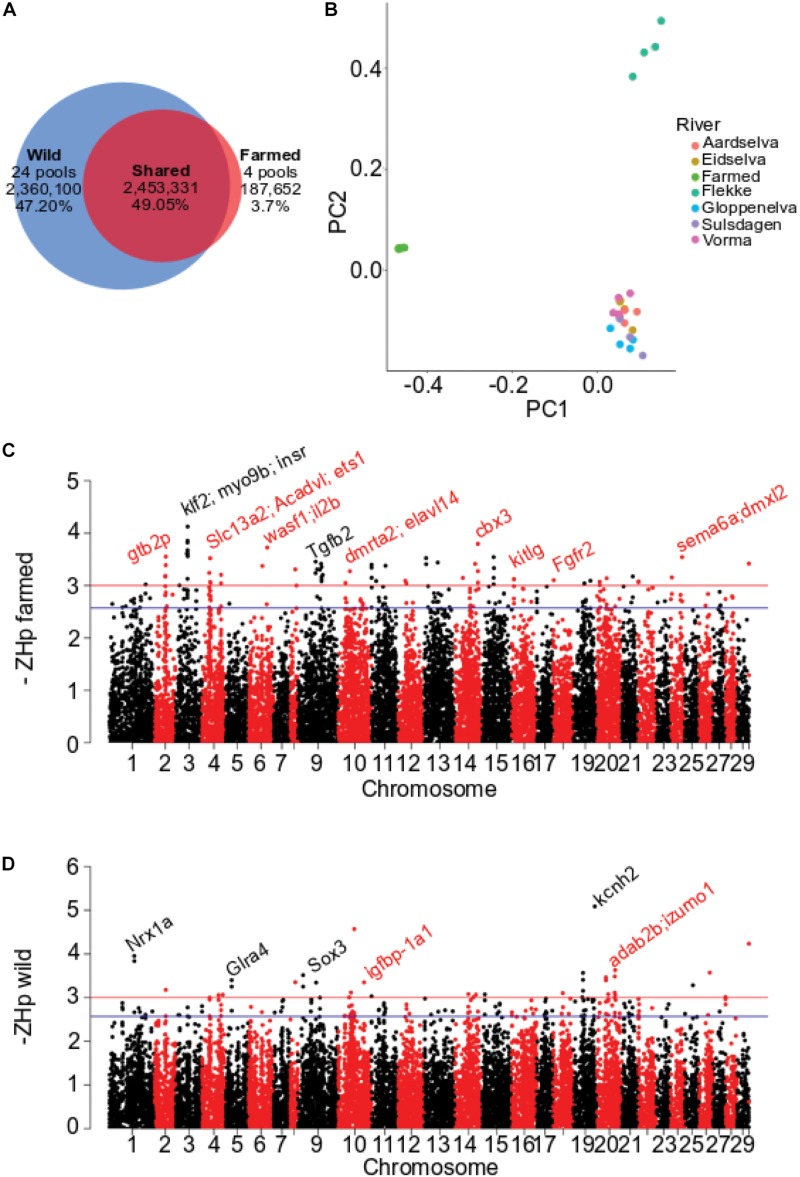
Distribution of pooled heterozygosity (*ZH*p) for Dataset 1. **(A)** Number and proportion of SNP shared between farmed and wild European Atlantic salmon. **(B)** Genomic relationship assessed using PCA of 28 pooled samples in Dataset1. PC1 explains 1.59% and PC2 1.13% of total variability. **(C)** Genome-wide plot of -*ZH*p estimated for farmed fish. **(D)** Genome-wide plot of -*ZH*p estimated for farmed fish.

To commence analysis of domesticated Atlantic salmon, four pools of genomic DNA containing 22 animals per pool were each sequenced to 20 – 24 fold coverage (Dataset 1, Table 1). These farmed fish have founders originating from ancestral populations of both the Norwegian and Scottish Atlantic clade of wild Atlantic salmon. We therefore selected previously published whole genome sequence data from wild Norwegian populations for comparison (Ayllon et al., 2015) (Table 1 and Supplementary Table S1). Variant calling across all pools identified 5,001,083 high-quality SNPs. Around half were independently identified in both the wild and domestic DNA pools (2.45 M of 49%, Figure 1A). A large proportion of the SNPs were found only in wild Atlantic salmon (2.36 M or 47%) while a comparatively small number (187,652 or 3.7%) were specific to farmed fish (Figure 1A and Supplementary Figure S1). Comparison against the available protein coding gene annotation for Atlantic salmon (Lien et al., 2016) revealed less than 1% of variants were in exons (232,030). The majority of exonic SNPs were synonymous substitutions (116,228, Supplementary Table S2). Prior to assessing patterns of variation that characterized farmed and wild populations, we checked the genomic relationship between pools using PCA and allele frequency divergence using *F*_ST_ (Figure 1B and Supplementary Figure S2). The pools of farmed fish clustered together and separately from wild populations using both PC1 and PC2 (Figure 1B and Supplementary Figure S2). The population wise *F*_ST_ values were consistent with the PCA analysis, with the highest values (0.052 – 0.073) found in pairwise comparison of farmed pools versus wild pools. The reduction in SNP frequency detected within the farmed pools, together with their divergence from wild populations, suggested Dataset 1 and the methods applied to identify genomic variation were well suited to subsequent analysis.

### Genomic Regions Putatively Under Selection Within Farmed or Wild Populations

Genomic regions impacted by positive selection are likely to display reduced heterozygosity in response to selection for beneficial alleles. To search for regions exhibiting depressed polymorphism, or an excess of homozygosity, pooled whole-genome sequence data (Dataset 1) was analyzed in 150 kb genomic windows using the *H*p metric (Rubin et al., 2010, 2012) (Supplementary Figure S3). When applied to genome data from farmed fish, 71 windows were identified as outliers from the 29,614 tested genome wide (*ZH*p < −3, *p*-val = 0.001; Figure 1C and Supplementary Table S3). The windows contained a small number of genes previously identified by genome scans for selection or with interesting functional roles. A prominent example is *kitlg*, a target of selection in multiple livestock species associated with melanocyte migration and pigmentation changes (Wilkinson et al., 2013; Qanbari et al., 2014). The same *ZH*p analysis of wild populations identified only 37 outlier windows (*ZH*p < −3, *p*-val = 0.001) (Figure 1D). The collection of genes contained within the outlier windows is given in Supplementary Table S4. The total number of windows that displayed significantly elevated homozygosity (71 and 37) is low when compared to similar studies conducted in livestock species. For example, analysis for domestic dog (Axelsson et al., 2013), chicken (Rubin et al., 2010), and pig (Rubin et al., 2012) all revealed generally higher rates of outlier window detection with more extreme –*ZH*p values than observed here. This likely reflects the very recent domestication of Atlantic salmon, in comparison to domesticated livestock and companion animals that have vastly different population histories characterized by much longer periods of artificial selection following domestication events that may have commenced more than 10,000 years ago (Larson et al., 2005).

### Selection Sweeps Identified by Comparison Between Domestic With Wild Populations

**FIGURE 2:**
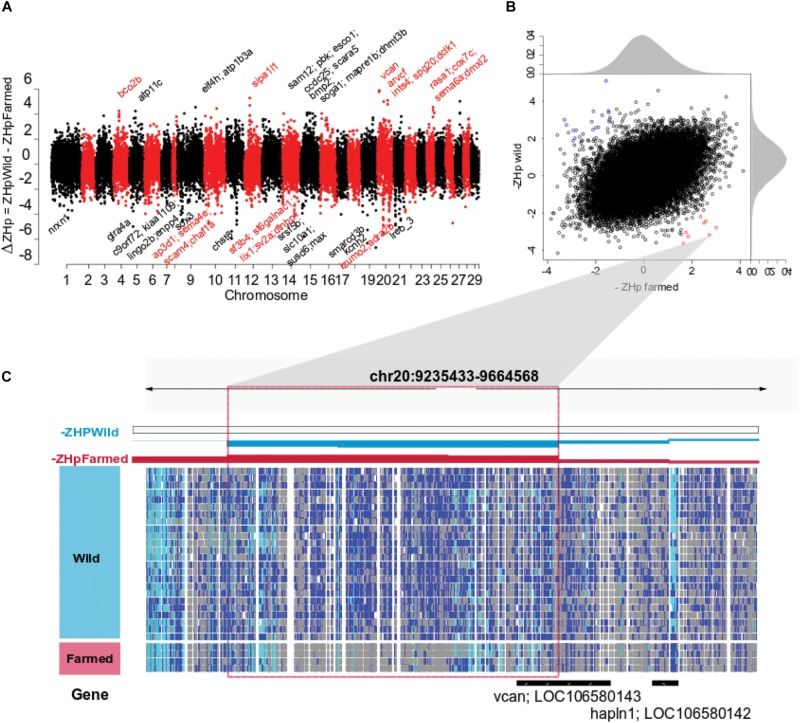
Selective sweep analysis for Dataset 1. **(A)** Genome wide distribution of Δ*ZH*p derived using both farmed and wild fish. Positive values indicate regions putatively under positive selection in farmed animals. **(B)** The –*ZH*p value for each genomic window was plotted for both farmed (*x* axis) and wild populations (*y* axis) to show their correlation (*r*^2^ = 0.457) and outlier behavior. Windows indicated in red have elevated homozygosity in farmed Atlantic salmon pools compared to wild (*p*-val ≤ 0.05) while windows indicated in blue are the opposite (*p*-val ≤ 0.05). **(C)** A 430 kb region of Atlantic salmon chromosome 20 is shown spanning two windows exhibiting the highest positive Δ*ZH*p values detected genome wide (5.79 and 5.89, Table 2). Genotypic status is shown as separate horizontal tracts for 20 pools of wild fish and 4 pools of farmed fish using the integrated genome viewer (IGV). SNP polymorphism within a pooled sample is indicated using dark blue. SNP homozygosity within a pool is indicated using either gray (where the fixed allele was the same as the reference genome) or cyan (where the fixed allele was the non-reference allele).

We sought to identify regions undergoing selection in a population specific manner by searching for regions with a marked difference in heterozygosity (Δ*ZH*p) between wild and farmed populations (Δ*ZH*p = *ZH*p_Wild_ – *ZH*p_Farmed_). Windows with increasingly positive Δ*ZH*p values exhibit elevated homozygosity in farmed fish while maintaining heterozygosity in wild populations, consistent with positive selection in response to domestication (Figure 2). This approach identified surprisingly few regions as *ZH*p_Wild_ and *ZH*p_Farmed_ values were significantly positively correlated (*r*^2^ = 0.457, *p*-val < 2.2 10^–16^, Figure 2B). A total of 139 regions had Δ*ZH*p > 3 (Figure 2A and Supplementary Table S5). After adjustment for multiple testing (Δ*ZH*p ≥ 4.55, *p*-adj 0.05), only 8 windows were identified showing a strong reduction in heterozygosity in farmed fish compared with their wild counterparts (Table 2). The most extreme window (Δ*ZH*p = 5.89) contained Versican (*vcan)*, a gene involved in the control of neural crest cell migration (Dutt et al., 2006). This is of particular interest given a broad range of neural crest cell regulatory genes have demonstrated roles in domestication related traits in other animals (Wilkinson et al., 2013; Pendleton et al., 2018). The sweep region is located upstream of *vcan* where each of the four pools of farmed fish have sharply reduced heterozygosity across a region spanning > 100 kb (Figure 2C). The sweep is noticeably absent in any of the 20 pools evaluated containing wild fish, giving rise to the most extreme outlier region genome wide. A further 131 regions had Δ*ZH*p < −3 and are indicative of sweeps specific to the wild populations (Supplementary Table S6).

**TABLE 2:** Putative selective sweeps detected in farmed Atlantic salmon (Dataset 1).

**chr**	**Start**	**End**	***ZH*p_dom_**	***ZH*p_Wild_**	****Δ***ZH*p**	**Alias**	**Genes product description**	**Function**	**References**	**Other species**
20	9375001	9525000	−2.72	3.18	5.89	Vcan	LOC106580143 versican core protein-like	Cell adhesion, proliferation, migration. Neural crest cells migration. Wound healing	Sotoodehnejadnematalahi and Burke, 2013	**Great apes** (Cagan et al., 2016) **Human** (Pääbo, 2014)
20	9300001	9450000	−3.00	2.79	5.79	–	–			
**12**	**27975001**	**28125000**	**−1.64**	**3.64**	**5.28**	**Sipa1l1**	**LOC106564813 uncharacterized LOC106564812 signal-induced proliferation-associated 1-like protein**	**Contributes to the regulation of dendritic spine morphogenesis**	Maruoka et al., 2005; Chutabhakdikul and Surakul, 2013	**+ Sheep** (Fariello et al., 2014) **Human** (Racimo, 2016; Peyrégne et al., 2017)
**20**	**53625001**	**53775000**	**−1.84**	**3.26**	**5.10**	–	**LOC106580979 LOC106580980 LOC106580981 olfactory receptor 1D2-like LOC106580982 olfactory receptor 6N1-like**	**Neuronal response that triggers the perception of a smell**		
27	26325001	26475000	−2.50	2.53	5.02	–	–			
**20**	**32775001**	**32925000**	**−2.57**	**2.44**	**5.00**	**arvcf**	**LOC106580542 LOC106580487 armadillo repeat protein deleted in velo-cardio-facial syndrome-like**	**Neurocognitive and neuroanatomical, craniofacial morphology**	Cho et al., 2011; Tran et al., 2011; Sim et al., 2012	**+ Dog breeds** (Persson et al., 2016)
**20**	**53700001**	**53850000**	**−1.74**	**3.04**	**4.79**	**gsonmt000 21581001 gsonmt 00046282001 ints4 spg20 dckl1 panx1**	**LOC106580981 olfactory receptor 1D2-like; LOC106580982 olfactory receptor 6N1-like; LOC106580972; LOC106580980; LOC106580973 40S ribosomal protein S3-like; LOC106580983 mth938 domain-containing protein-like; LOC106580975; LOC106580974 spartin-like%2C transcript variant; LOC106580976 serine/threonine-protein kinase DCLK1-like%2C transcript variant**	**Spg20; synaptic growth and neuronal survival, linked to spastic paraplegia DCLK1: neuronal migration, neurogenesis Panx1: inflammation; myoblast differentiation; neural precursor and maintenance**	Nahm et al., 2013; Butler et al., 2016; Dardour et al., 2017 Nawabi et al., 2015; Wicki-Stordeur et al., 2016; Langlois and Cowan, 2017	+ (*) DCX: (Huang et al., 2015)
9	95175001	95325000	−2.36	2.20	4.55	Eif4h gsonmt 00055384001 atp1b3a	if4h Eukaryotic translation initiation factor 4H LOC106612405 E3 ubiquitin-protein ligase rififylin-likeLOC106612413 sodium/potassium-transporting ATPase subunit beta-3-like	Atp1b3:osmoregulation		

*150 kb regions chr, start, end, *ZH*pvalue in Atlantic salmon farmed pools, *ZH*p scores in Atlantic salmon wild pools, Δ*ZH*pPools= *ZH*p_Wild_ – *ZH*p_*Farmed*,_ Overlapping gene Alias as from SalmoBase Gene Browser track, Overlapping Genes product LOC identifier and gene product description. Regions in bold confirmed in an independent farmed and wild set (Dataset 2).*

### Sweep Regions Assessed Using an Independent Dataset

**FIGURE 3:**
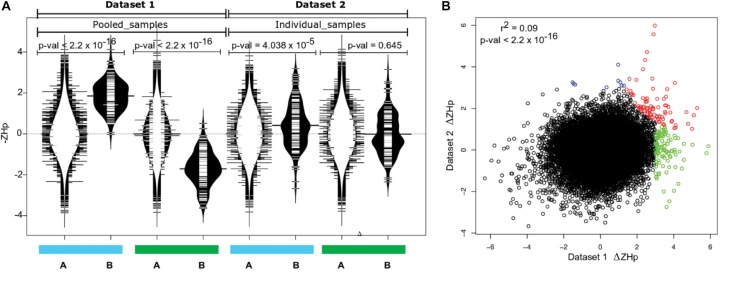
Common signals of selection in two independent datasets. **(A)** Putative selection sweeps in farmed fish from Dataset 1 (139 regions *p*-val < 0.001) contain higher fixation than expected in farmed but not in wild individuals in Dataset 2. The blue and green bars denote farmed and wild fish data respectively. The distribution of –ZHp values is shown for all windows genome wide (A, distribution plots with white interior) and separately only for the 139 windows putatively under selection in farmed salmon within Dataset 1 (B, distribution plots with black interior). **(B)** Correlation between Δ*ZH*p for Dataset 1 and Dataset 2 identified genomic windows with outlier behavior in one or both datasets (*r*^2^ = 0.09; *p*-val < 2.2 × 10^–16^). Windows colored red denote significant positive Δ*ZH*p in both Dataset 1 and Dataset 2 (Supplementary Table S7). Green windows were significantly positive only within Dataset 1 and blue only within Dataset 2. Outliers with significantly negative Δ*ZH*p were not colored but are listed in Supplementary Table S8.

The putative selection sweeps identified using Dataset 1 provide an interesting basis for discussion, but verification within independent populations are required to provide strong evidence that they are the result of domestication. The wild populations assayed may not be strongly enriched for ancestor fish recruited to initiate the farmed populations, or the pool-seq approach may introduce bias, each leading to the potential for the detection of false positive selection sweeps. Therefore, we repeated aspects of the analysis using Dataset 2, an independent collection of individually sequenced farmed and wild European derived Atlantic salmon (Dataset 2, Table 1). Variant calling for Dataset 2 yielded 2,251,066 SNPs, before genome-wide *ZH*p scores were calculated as performed for Dataset 1. Comparative analysis revealed the collection of outlier windows identified in Dataset 1 (139 genomic windows with Δ*ZH*p ≥ 3, *p*-val = 0.001) displayed increased homozygosity compared with their genome-wide mean within the commercially farmed salmon in Dataset 2 (Wilcoxon test, *p*-val = 4.038 × 10^–5^). Importantly, this was not the case within the wild salmon individuals in Dataset 2 (Figure 3A). This strongly suggested that the sweeps identified using Dataset 1 are enriched for true selection sweeps and are unlikely to be entirely due to drift or the result of the populations used in the genome wide comparisons. We next sought to identify the number and distribution of genome windows that displayed significantly positive Δ*ZH*p values within the farmed fish populations in both Datasets 1 and 2 (Figure 3B). This yielded 81 regions (*p*-adj < 0.05) with significantly reduced heterozygosity in farmed salmon compared to wild fish in both datasets (Supplementary Table S7). Investigation of the genes contained in these windows revealed clear relevance to domestication and/or genes with established roles in phenotypic variation. At least three genes are related to neurogenesis and neural function suggesting a relationship with behavioral traits (*arvcf*, *sema6a*, and *erbb4*; Figures 2, 3 and Supplementary Table S7). Others relate to neural crest cell migration (*txbl-a*) and olfactory mechanisms (*id2-like*, *6nl-like*), both of which have plausible roles in processes associated with domestication and adaptation to a captive environment.

### Sweep Regions in North American Derived Farmed Atlantic Salmon

**FIGURE 4:**
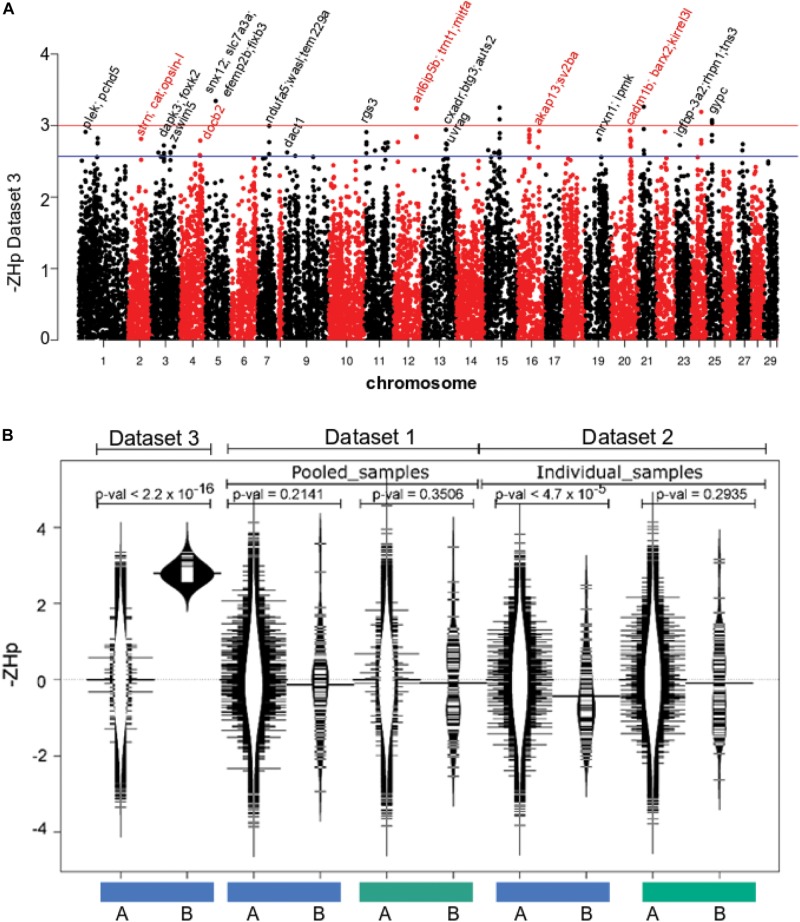
Selective sweep analysis in farmed Atlantic salmon of North American origin. **(A)** The genome wide distribution of *ZH*p in farmed Atlantic salmon from Tasmania (Dataset 3). **(B)** Genome windows with elevated homozygosity in Dataset 3 do not exhibit similarity increased homozygosity in either Datasets 1 or 2. The blue and green bars denote farmed and wild fish data respectively. The distribution of –ZHp values is shown for all windows genome wide (A, distribution plots with white interior) and separately only for the 79 windows putatively under selection in farmed Atlantic salmon within Dataset 3 (B, distribution plots with black interior).

The final component of the analysis sought to identify evidence for parallel selection sweeps founded independently within farmed stocks established from both European and North American wild populations. Dataset 3 comprised 19 genomes of farmed fish from Tasmania that originate from Canadian stocks (Table 1). Variant calling yielded 7,623,909 high quality SNPs before heterozygosity was assessed as *ZH*p and plotted as a function of genomic location (Figure 4A). Few genomic regions had evidence for either mild (79 windows, –*ZH*p > 2.57; *p*-val < 0.01) or strong increases in homozygosity (9 windows, –*ZH*p > 3; *p*-val < 0.001, Table 3). The number of identified outliers was lower than detected within the European derived farmed population in Dataset 1 (Figure 4A). The most extreme outlier window was found on Ssa15, and spanned a region containing more than 10 genes making it difficult to identify the likely target(s) of selection. We assessed if the small collection of outlier regions identified from Atlantic salmon in Tasmania (79 windows with –*ZH*p > 2.57) showed evidence for increased homozygosity within the farmed European fish in either Datasets 1 or 2. No evidence was present (Figure 4B), suggesting little or no overlap appears to be present between putative sweep regions found in European and North American derived farmed populations. On the basis of the analysis performed here, parallel selection events do not appear to be a significant factor shaping patterns of genetic variability.

**TABLE 3:** Candidate Selective sweeps detected in North American Atlantic Salmon samples from Tasmania (Dataset 3).

**chr**	**Start**	**End**	***ZH*p**	**Alias**	**Genes product description**
5	32325001	32475000	3.34	ubl3 atl3 dipa fibp slc7a3a snx12 fosl2 prs23 efemp2b foxn3 cof2	ubl3; ubiquitin-like 3LOC106604707; serine protease 23-like%2C transcript variant LOC106604709; atlastin-3-likeLOC106604711; protein YIF1A-like%2C transcript variant LOC106604713; coiled-coil domain-containing protein 85B-like%2C transcript variant LOC106604715; acidic fibroblast growth factor intracellular-binding protein-likesnx12; sorting nexin 12%2C transcript variant slc7a3; solute carrier family 7 (cationic amino acid transporter%2C y+ system)%2C member 3%2C transcript variant LOC106604708; cofilin-2-likeLOC106604710; forkhead box protein N2-like%2C transcript variant LOC106604712; fos-related antigen 1-like%2C transcript variant LOC106604716; EGF-containing fibulin-like extracellular matrix protein 2%2C transcript variant
21	17625001	17775000	3.26	i20ra orf2p i10r2 crfb2 btn1a1 crfb1a	20ra; Interleukin-20 receptor alpha chainLOC106581740; uncharacterized LOC106581740%2C transcript variant LOC106581741; interleukin-10 receptor subunit beta-likeLOC106581720; CD276 antigen homolog%2C transcript variant LOC106581739; uncharacterized protein C21orf62-like%2C transcript variant
15	45075001	45225000	3.25		LOC106571716; uncharacterized protein K02A2.6-likeLOC106571620; C-C chemokine receptor type 6-like
12	72000001	72150000	3.24	gsonmt00065759001 arl6ip5b trnt1 nft-vtr mitfa gsonmt00065604001	
24	30900001	31050000	3.19	cep78 naa35 gnaq vps13a agtpbp1 gna14	naa35; *N*(alpha)-acetyltransferase 35%2C NatC auxiliary subunit %2C transcript variant LOC106585634; guanine nucleotide-binding protein subunit alpha-14-likeLOC106585689; guanine nucleotide-binding protein G(q) subunit alphacep78; centrosomal protein 78 kDa %2C transcript variant golm1; golgi membrane protein 1vps13a; vacuolar protein sorting 13 homolog A (*S. cerevisiae*) %2C transcript variant agtpbp1; ATP/GTP binding protein 1%2C transcript variant
15	45150001	45300000	3.09		LOC106571620; C-C chemokine receptor type 6-like
25	16725001	16875000	3.07	gypc	LOC106586280; glycophorin-C-like%2C transcript variant
25	16800001	16950000	3.05	Unknown	LOC106586279; contactin-associated protein-like 5
25	17100001	17250000	3.03	gsonmt00061935001	

## Discussion

The primary objective of the study was to deepen our understanding of how the captive farming environment and artificial selection have impacted patterns of variation across the genome of Atlantic salmon. A number of previous studies have addressed this question, resulting in the identification of largely non-overlapping genomic regions and gene sets (Bourret et al., 2011; Vasemägi et al., 2012; Martinez et al., 2013; Mäkinen et al., 2015; López et al., 2019b). We designed this experiment to be differentiated from earlier work in at least two important ways. First, we exploited the availability of whole genome data which holds the promise of increased precision to detect sweeps over low and medium-density SNP arrays. Genome sequencing data also opens the possibility of detecting functional variants that directly underpin trait variation. Secondly, the experimental design incorporated analysis of multiple independent populations to address the issue of false positive sweep signals. This was important given the farmed and wild population comparisons used were not optimal. Specifically, ancestors of the farmed fish were not sourced from the rivers sampled to generate the wild fish genome data. This leaves open the possibility that phylogenetic structure may contribute to the observed patterns of SNP variation in addition to selection following domestication. The finding that outlier regions with reduced heterozygosity in Dataset 1 also showed significantly lower heterozygosity in Dataset 2 provided confidence the shared regions have biological validity. Analysis of the genes present in putative sweep regions revealed a number have established roles relevant to domestication, with functions relating to brain function and behavioral traits prominent. This is consistent with findings from other species such as rabbit which have a comparatively short domestication history compared with most livestock species (Carneiro et al., 2014). Reduction in fear and changes to social behavior are cases in point (Jensen, 2014). In this study, *arvcf* was identified in a sweep region on Ssa20 in both Dataset 1 and 2. The gene was previously associated with dog sociability to humans (Persson et al., 2016), while a paralog of *dclk1* (doublecortin like) termed *dcx* (doublecortin) was found in a separate sweep on chromosome 20 that has been associated with tameness in foxes (Huang et al., 2015). A selective sweep on Ssa24 spans *sema6a* that is under selection in domestic cattle (Qanbari et al., 2014). It has been directly associated with exploration behavior, with knockout mice expressing marked behavioral differences (Håkansson et al., 2017). A recent study has also identified the related gene *sema6b* as a candidate underlying selection in farmed Atlantic salmon (López et al., 2019b). It has a major role in axon guidance and both peripheral and central nervous system development. A fourth behavioral gene, *erbb4*, was identified on Ssa21 that has evidence of selection in domestic cattle (Qanbari et al., 2014). Expression changes in *errb4* in mice regulated fear and mania-like behaviors (hyperactivity, reduced anxiety and depression and increased sucrose preference) (Chen et al., 2017; Cao et al., 2018). Each of these findings suggest behavioral adaptations to the farmed environment, including altered response to threats, and differences in aggression and feeding behavior may be an early consequence of domesticating Atlantic salmon (Huntingford, 2004; Glover et al., 2017). Interestingly, a selective sweep on Ssa20 contained two olfactory receptor genes (olfactory receptor *id2-like* and *6n1-like*) (Table 2). The olfactory system plays a key role in the ‘homing’ instinct of wild salmon (Nevitt and Dittman, 2004), and multiple studies have demonstrated that offspring of domesticated Atlantic salmon display reduced survival in the marine phase of early life (McGinnity et al., 2003; Skaala et al., 2019). The homing instinct is no longer relevant in the farmed environment and may be associated with behaviors or physiology with negative consequences for survival or growth in culture. Further, olfactory receptors are associated with domestication selection in several other species, including dogs (Chen et al., 2012), cats (Montague et al., 2014), and pigs (Amaral et al., 2011). The gene *sipa1l1* identified in a sweep region on Ssa12, which is involved in the regulation of dendritic spine morphogenesis, and therefore, a key role in synaptic process, has also been identified as a gene potentially subjected to domestication and selection in two independent studies in Atlantic salmon (López et al., 2019a, b). The final gene of interest is versican (*vcan*), which displayed the most extreme outlier behavior in Dataset 1. The gene acts to control neural crest cell migration (Landolt et al., 1995; Dutt et al., 2006), a fundamental developmental process whereby multipotent stem cells emerge from the neural crest before differentiating into a wide variety of cell types and physiology. They play a central role in body conformation, pigmentation, neuronal changes and craniofacial development, each of which are often impacted in animals by domestication. A recent study that compared village dog and gray wolf genomes illustrates this well, as a range of genes were implicated in domestication that have clear roles in neural crest cell pathways and their control (Pendleton et al., 2018). These findings have given rise to the hypothesis that neural crest cell migration is a key mechanism by which domestication acts to alter multiple animal characteristics (Wilkinson et al., 2013). Our results indicate this may also by the case in Atlantic salmon, although it remains unclear by what mechanism. It is also noteworthy *vcan* was significantly associated to human lean body mass in meta-analysis of more than sixty thousand individuals (Zillikens et al., 2017), suggesting it may have undergone change as farmed Atlantic salmon are selectively bred for improved growth rate and weight.

Independent domestication events, whereby farmed fish stocks were founded from either European or North American wild stocks, offered the opportunity to search for evidence of parallel selection sweeps. It is important to note that earlier analysis of genome sequence comparing European and North America Atlantic salmon revealed them to be highly genetically divergent (Kijas et al., 2017). Comparing outlier regions in this study (from Dataset3 with those from either Datasets 1 or 2) failed to detect an overlap. This is consistent with the latest findings on parallel evolution in Atlantic salmon from López et al. (2019a). The authors used a 200K SNP array and found only four regions of overlap between populations with North American and European origins. This suggests parallel genetic changes resulting from domestication are rare, and our results in this study obtained using whole genome sequencing datasets provide additional evidence. This may arise due to differences in nutrition, management or environmental conditions. Alternatively, largely distinct sets of genes may have undergone change between populations and result in similar phenotypic changes to complex traits. In addition, it is worth noting that unlike European farmed Atlantic salmon populations under active selection for economic traits since the 1970s, family selection commenced for the Tasmanian population in 2005. Even where artificial selection has acted to promote a trait in common, for example growth rate, the growing body of evidence suggest selection has acted on different subsets of genes.

## Data Availability Statement

The datasets generated for this study can be found in NCBI PRJNA293012, PRJEB10744, SRP059652, PRJNA403334, and PRJNA614520.

## Ethics Statement

Ethical review and approval was not required for the animal study because Animals used in this study were part of the commercial operations of Salmon Enterprises of Tasmania Pty. Ltd. Their use was in accordance with authorized management practices of the company and compliant with the Tasmanian Animal Welfare Act (1993) which is under the jurisdiction of Biosecurity Tasmania, Department of Primary Industries, Parks, Water and Environment. Under this Act, those animals that are expressly killed for purposes other than research, such as abattoir specimens, do not need specific approval of an Animal Ethics Committee and that was the case for this study. Written informed consent was obtained from the owners for the participation of their animals in this study.

## Author Contributions

MN-S, RH, and JK conceived the study and contributed to the experimental design. RH and BE contributed to animal samples, genome data or both. MN-S, SM, and JK performed the analysis. MN-S and JK wrote the manuscript. All authors contributed to the final version.

## Conflict of Interest

BE was employed by Salmon Enterprises of Tasmania (Saltas) and Tassal. The remaining authors declare that the research was conducted in the absence of any commercial or financial relationships that could be construed as a potential conflict of interest.

## Footnotes

1 http://broadinstitute.github.io/picard/

2 https://www.cog-genomics.org/plink2
