# Supplementary material for: Changed Patterns of Genomic Variation Following Recent Domestication: Selection Sweeps in Farmed Atlantic Salmon
Source: Front Genet. 2020 Apr 3;11:264. doi: 10.3389/fgene.2020.00264 (PMC7147387; doi:10.3389/fgene.2020.00264)
Supplement: Supplementary file 9 [file Presentation_1.pdf]

Supplementary Information

Signatures of selection in farmed Atlantic salmon reveal rapid evolution of behavioural genes

Marina Naval-Sanchez[1], Sean McWilliam[1], Brad Evans[2], Jose Manuel Yanez[3], Ross D. Houston[4] and James W. Kijas[1]

Affiliations

[1]*CSIRO Agriculture & Food, 306 Carmody Rd., St. Lucia, Brisbane, QLD 4067, Australia*

[2]Salmon Enterprises Of Tasmania Pty. Limited (SALTAS), Wayatinah, Tasmania, Australia

[3] *The Roslin Institute and Royal (Dick) School of Veterinary Studies, University of Edinburgh, Midlothian, UK*

[4] *Faculty of Veterinary and Animal Sciences, University of Chile, Av. Santa Rosa, 11735, La Pintana, Santiago, Chile*

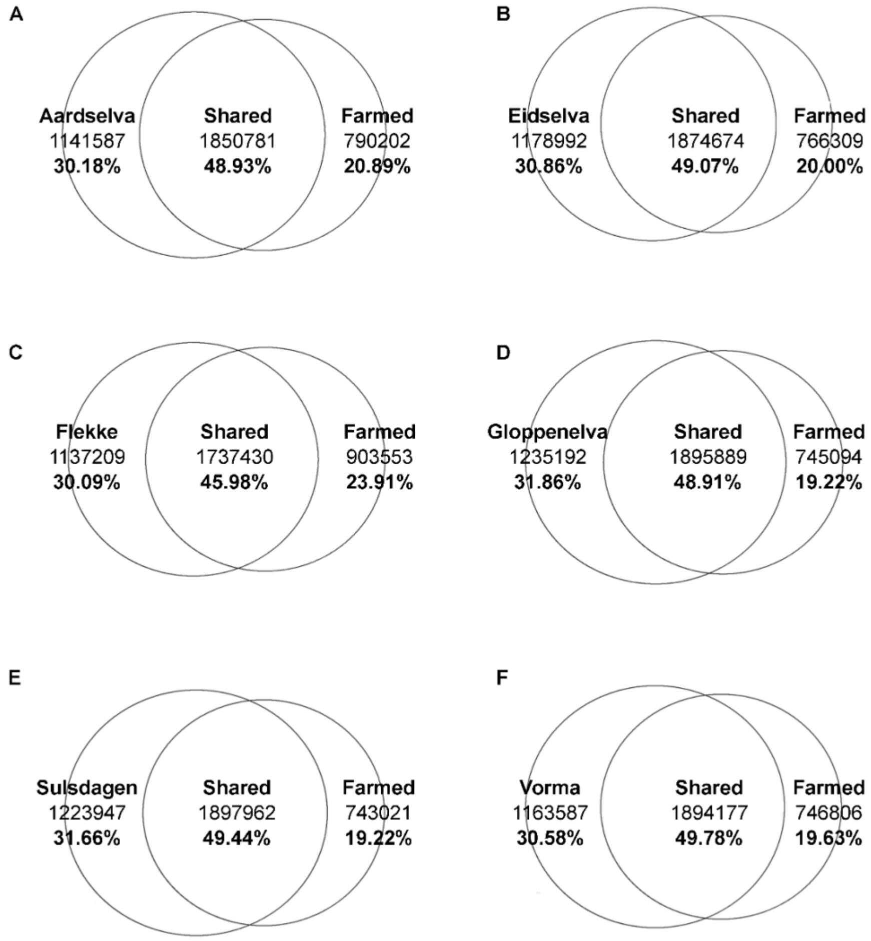

Supplementary Figure S1. Number of SNPs shared across the European Atlantic salmon farmed population and four wild populations sourced from different Norwegian rivers (A) Aardeslva (B) Eidselva (C) Flekke (D) Gloppenelva (E) Sulsdagen (F) Vorma. Despite farmed populations having higher number of individuals per pool (22-24) and higher sequencing coverage (22X) compared to wild populations (20 individuals per pools and 16X coverage), they present lower number of private SNP.

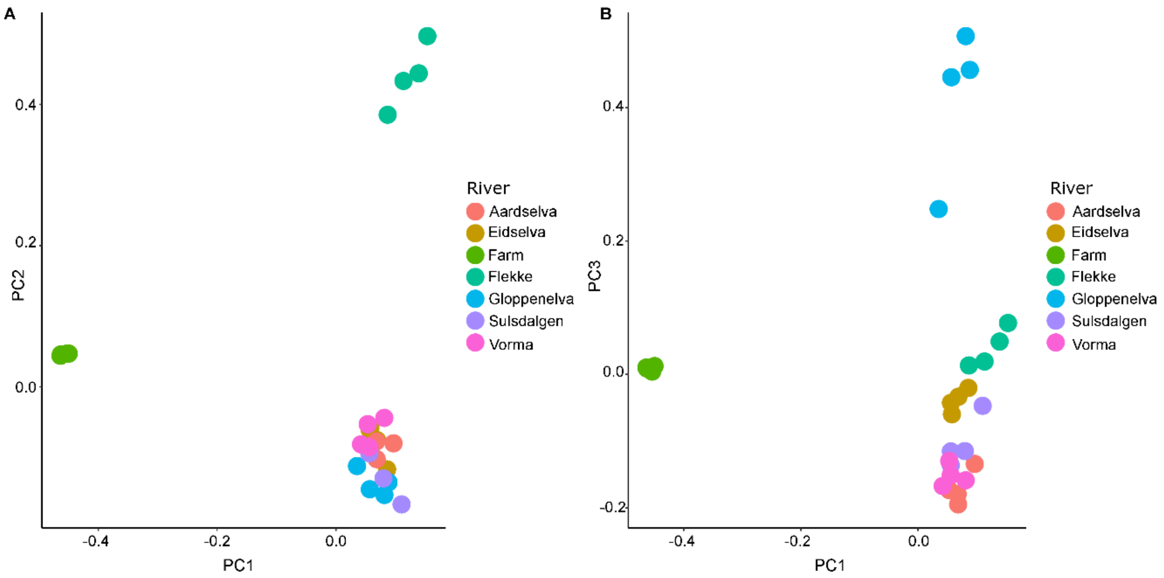

Supplementary Figure S2. Genomic relationships across Atlantic salmon pooled samples by PCA. (A) PC1 and PC2 for all pools in Dataset 1. PC1 explains 1.59% and PC2 1.13% of total variability. **(B)** PC1 and PC3 for the same pools. PC3 explains 0.93% of total variability.

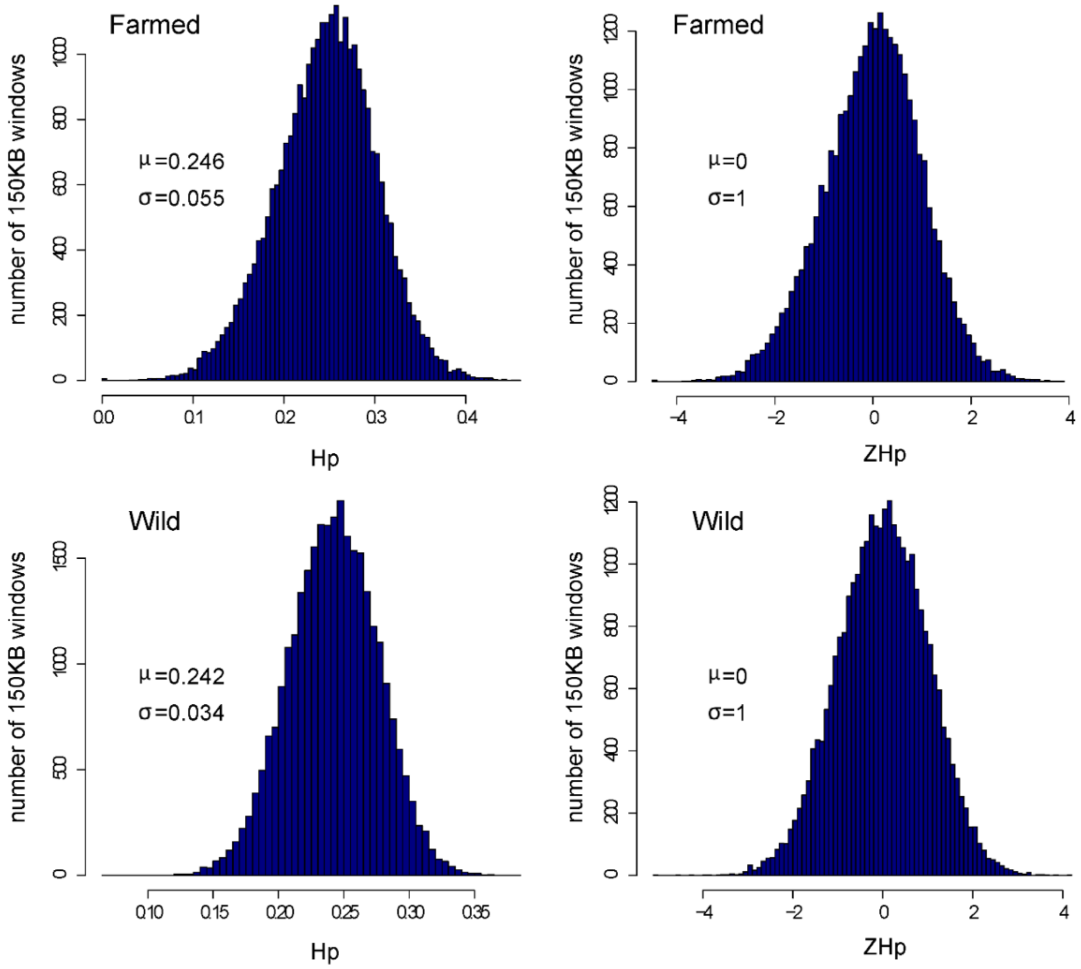

Supplementary Figure S3. Distribution of heterozygosity values (H_p, left hand side panels) and their Z-transformations (ZH_p, right hand side panels) for either farmed fish (top panels) or wild Atlantic salmon (bottom panels) in Dataset 1. Genome windows (150 kb) were excluded that contained less than 20 SNP, leaving 29,614 windows in farmed fish and 29,688 windows in wild fish. *ZH*p <=3 is the threshold used to identify potential selective sweeps p-val <= 0.001.

Supplementary Table S1. Summary Mapping Statistics for pooled samples in Dataset 1.

Supplementary Table S2. Annotation of SNP Identified using Dataset1.

Supplementary Table S3. Outlier regions and associated genes detected in farmed fish from Dataset1 with - ZHp > 3.

Supplementary Table S4. Outlier regions and associated genes detected in wild fish from Dataset1 with - ZHp > 3.

Supplementary Table S5. Genomic windows with outlier behaviour for ΔZHp values from farmed fish in Dataset1.

Supplementary Table S6. Genomic windows with outlier behaviour for ΔZHp values from wild fish in Dataset1.

Supplementary Table S7. Genome widows with significantly positive ΔZHp values in both Datasets 1 and 2.

Supplementary Table S8. Genome widows with significantly negative ΔZHp values in wild fish from both Datasets 1 and 2.
